# Expression of recombinant Omp18 and MOMP of *Campylobacter jejuni* and the determination of their suitability as antigens for serological diagnosis of campylobacteriosis in animals

**DOI:** 10.14202/vetworld.2023.222-228

**Published:** 2023-01-30

**Authors:** Sergey Borovikov, Kanat Tursunov, Alfiya Syzdykova, Ainagul Begenova, Alfira Zhakhina

**Affiliations:** 1 Department of Microbiology and Biotechnology, Faculty of Veterinary and Animal Husbandry Technology, S. Seifullin Kazakh Agro Technical University, 010000, Astana, Kazakhstan; 2 Laboratory of Immunochemistry and Immunobiotechnology, National Center for Biotechnology, 010000, Astana, Kazakhstan

**Keywords:** *Campylobacter jejuni*, campylobacteriosis, diagnostics, outer membrane proteins, recombinant antigens

## Abstract

**Background and Aim:**

Campylobacteriosis causes gastrointestinal tract lesions in adults and children and may result in severe complications. The primary sources of infection are infected animals and animal products. Immunochemical methods effectively diagnose intestinal infections but require highly specific antigens to detect their antibodies. This study aimed to obtain two recombinant immunogenic antigens of *Campylobacter* *jejuni*, an outer membrane protein with a molecular weight of 18 kDa (Omp18) and the major outer membrane protein (MOMP) with a molecular weight of 45 kDa, and evaluate their suitability for the serological diagnosis of campylobacteriosis using immunochromatographic assay (ICA).

**Materials and Methods:**

The *C. jejuni* Omp18 and MOMP gene sequences were synthesized *de novo* (Macrogen, Korea) and cloned into the pET32 expression plasmid. Using these genetic constructs, electrocompetent cells of the *Escherichia coli* BL21 strain were transformed and cultured under various conditions. Antigens were purified and refolded using metal affinity chromatography. The properties of the purified proteins were studied by western blotting, liquid chromatography with tandem mass spectrometry, and enzyme-linked immunosorbent assay (ELISA).

**Results:**

We developed two recombinant *E. coli* BL21 cells producing rOmp18 and Recombinant MOMP (rMOMP) antigens with molecular weights of 36 and 64 kDa, respectively. Amino acid sequence analysis of the obtained antigens showed complete homology with the reference sequences in the PubMed NCBI database. Western blotting using positive-control sera demonstrated the specificity of the recombinant antigens. The results of ELISA with 94 bovine sera showed the interaction of recombinant antigens with specific antibodies.

**Conclusion:**

The obtained rOmp18 and rMOMP antigens can detect antibodies in the serum of infected or recovered animals and can be used to develop ICA.

## Introduction

Campylobacteriosis is one of the main causes of gastrointestinal tract diseases worldwide [1]. The most dangerous causative agents of campylobacteriosis are *Campylobacter jejuni* and *Campylobacter coli* [2, 3]. In addition to diarrhea, *C. jejuni* causes reactive arthritis, pancreatitis, and carditis. Some strains of *C. jejuni* can stimulate the production of antibodies that react with peripheral nerve myelin, causing Guillain-Barré syndrome [4–6]. The lack of specificity of clinical signs in campylobacteriosis places special demands on diagnostics, delaying timely therapeutic intervention for the disease. Considering the difficulties associated with the cultivation of *Campylobacter*, molecular methods such as real-time polymerase chain reaction (PCR) and enzyme-linked immunosorbent assay (ELISA) have been developed and commercialized [7–9]. At present, immunochromatographic assays (ICAs), which are easy to use and rapid, are widely used [10–12]. However, despite the high sensitivity of ICAs, their specificity is significantly inferior to bacteriological analysis [4].

A possible method to increase the specificity of ICA is by utilizing immunogenic bacterial membrane proteins. Several surface antigens of Campylobacter species have been characterized genetically and immunologically. These include flagellin, *C. jejuni* PEB1 protein, membrane-associated protein A, and Omp18 [13, 14]. Major outer membrane proteins (MOMPs) are involved in the regulation of membrane permeability to various small molecules. *Campylobacter jejuni* MOMP is an immunodominant protein with variable regions that can generate species-specific monoclonal antibodies (mAbs) [15, 16].

This study aimed to develop recombinant Omp18 and MOMP (rOmp18 and Recombinant MOMP [rMOMP]) proteins of *C. jejuni*, with the corresponding gene sequences synthesized *de novo*. The obtained recombinant proteins were analyzed by liquid chromatography with tandem mass spectrometry (LC-MS/MS) spectrometry, western blot, and ELISA. The sequence of the recombinant proteins and their affiliation to *C. jejuni* was confirmed by the Mascot program using SwissProt as the sequence database.

## Materials and Methods

### Ethical approval

All animal experiments were carried out with the permission of the Local Ethical Committee of S. Seifullin Kazakh Agro Technical University (Protocol No. 1 dated January 11, 2021).

### Study period and location

The study was conducted from September 2021 to June 2022. Recombinant proteins were obtained from the Laboratory of Immunochemistry and Immunobiotechnology of the National Center for Biotechnology. A sampling of blood sera from cattle was carried out from March to May 2022 on the farms of the Akmola and Karaganda regions of the Republic of Kazakhstan. Control sera were kindly provided by the National Veterinary Reference Center, Astana, Kazakhstan.

### Bacterial strains, plasmids, and proteins

*Escherichia coli* strains DH5α, BL21(DE3) (Novagen, Madison, WI, USA), plasmid vector pGEM-TEasy (Promega, Madison, WI, USA), pET28, and pET32 (Novagen) were used. *Escherichia coli* cells were grown in Luria-Bertani (LB) broth. Tag DNA Polymerase (Thermo Fisher Scientific, Waltham, MA USA) was used to screen *E. coli* colonies for expression plasmids.

### Gene design, cloning, transformation, and expression detection

The reference amino acid sequences of the Omp18 (VTQ54093.1) and MOMP (ABI29902.1) proteins were taken from the PubMed NCBI database (https://www.ncbi.nlm.nih.gov/protein/). The Omp18 and MOMP protein genes were codon-optimized for the *E. coli* K12 expression system using the VectorNTI program. The *Omp18* and *MOMP* genes were synthesized by Macrogen Inc. (Seoul, Korea). The synthesized genes were cloned into the pET32 expression plasmid. DNA fragments were excised from plasmids using *Nco*I and *Xho*I enzymes (Thermo Fisher Scientific) and Tango buffer in a water bath at 37°C for 2 h. The Hanahan method was used to transform cells of laboratory *E. coli* DH5a strains [17]. The transformation efficiency was 1 × 10^5^ transformants per 1 mg supercoiled DNA of the pGEM-TEasy plasmid. When cloning DNA fragments in plasmid vectors, counter selection methods on media with antibiotics and recombinant screening using isopropyl-b-D-1-galactopyranoside (IPTG) and X-gal were used to search for recombinants. Plasmid DNA purification was performed using the PureLink™ HiPure Plasmid Filter Midiprep Kit (Thermo Fisher Scientific) according to the manufacturer’s recommendations. Purified pET32/Omp18 and pET32/MOMP were used to transform electrocompetent BL21(DE3) cells, followed by the addition of Super Optimal broth with Catabolite repression and incubation for 50 min at 37°C, 150 rpm. After selecting positive colonies by PCR, protein expression was determined. For this, the cell culture in the exponential phase was induced with 0.2 mM IPTG and the expression was checked every 2 h.

### Isolation and purification of recombinant proteins

The cell pellet (0.9 g), obtained from 250 mL of cell culture, was resuspended in Tris-NaCl-EDTA buffer and sonicated using UP200S. The pellet, after centrifugation, was resuspended in buffer with 1 M urea, incubated for 30 min at 25°C, and centrifuged at 18,500 × *g* for 40 min. The pellet was then dissolved in buffer with 8 M urea, and sonicated. Recombinant proteins were purified using HisTrap HP 1 mL columns (Cytiva, Uppsala, Sweden) by immobilized metal ion affinity chromatography. A linear gradient of urea (8–0 M) was passed through the column with a final volume of 30 mL at a rate of 0.5 mL/min to refold the recombinant proteins. Proteins were eluted with a buffer supplemented with an imidazole gradient from 20 to 500 mM. We used a liquid chromatograph for fast purification (AKTA purifier 10, Amersham Biosciences, Uppsala, Sweden) of proteins. The protein concentration was determined by the Bradford method. Analysis of protein purification was performed by electrophoresis in 12% polyacrylamide gel in the presence of sodium dodecyl sulfate-polyacryl­amide gel electrophoresis (SDS-PAGE).

### Western blot

Electrophoresis of recombinant proteins was performed as described above, followed by their wet transfer to a nitrocellulose membrane using the Mini-PROTEAN Tetra cell system (BioRad, Hercules, CA, USA). For immunochemical identification of the desired antigens, the membrane was blocked with 1% Bovine Serum Albumin (BSA) for 1 h at 25°C. After washing with PBS-Tween 20 (PBS-TW), mAbs against 6His-tag (1:3000) was added and incubated for 1 h at 37°C. The membrane was then washed and secondary antibodies were added for 50 min at 37°C. After washing the membrane, the reaction was initiated by adding the substrate 4-chloronaphthol (Amresco, Solon, OH, USA). After the appearance of gray protein spots, the reaction was terminated by washing the nitrocellulose membrane in distilled water.

### Nanoscale liquid chromatography coupled to tandem mass spectrometry

Purified samples of recombinant proteins were separated on 12% SDS-PAGE followed by excision of the bands. A mixture of NH_4_HCO_3_ in acetonitrile (1:1) was added to the crushed fragments and incubated for 35 min at 37°C. After further processing to decrease the size of the gel fragments and reduce disulfide bonds, the gel fragments were subjected to protein digestion by the addition of trypsin and 50 mM NH_4_HCO_3_. After trypsinization, the mixture was desalted using a commercial kit (Ziptips Micro-C18, Millipore, Burlington, MA, USA). The peptides were separated by HPLC and analyzed by LC-MS/MS spectrometry using Acclaim™ PepMap™ RSLC column (Thermo Fisher Scientific). For matching the spectra in the SwissProt database, Mascot software was used.

### Enzyme-linked immunosorbent assay

For immobilization, 10 µg/mL recombinant proteins rMOMP and Omp18 (in 0.1 M NaHCO_3_, pH = 9) from *C. jejuni* were added in Nunc-Immuno™ MicroWell™ “Maxisorp” 96-well microplates (Nalge International, Rochester, NY, USA) and incubated for 60 min at 37°C. The microplates were blocked with 1% (w/v) BSA in PBS for 60 min at 37°C. Serum from recovered cows was double diluted in PBS and added to recombinant proteins and incubated for 60 min at 37°C. The microplates were washed with PBS-TW buffer, followed by the addition of a secondary antibody and further incubation for 60 min at 37°C. The microplates were then washed with PBS-TW and PBS followed by the addition of 3,3´,5,5´ tetramethylbenzidine substrate (ThermoFisher Scientific) (100 µL/well) and incubation for 15 min at room temperature. The reaction was stopped by adding 2 M H_2_SO_4_ and absorbance at 450 nm was measured using a microplate reader.

### Statistical analysis

Statistical analysis was performed using GraphPad Prism 9.4.1. (https://www.graphpad.com/updates/prism-941-release-notes), with Student’s p-value (p ≤ 0.05).

## Results

### Cloning, expression, and purification of recombinant proteins

**Figure-1:**
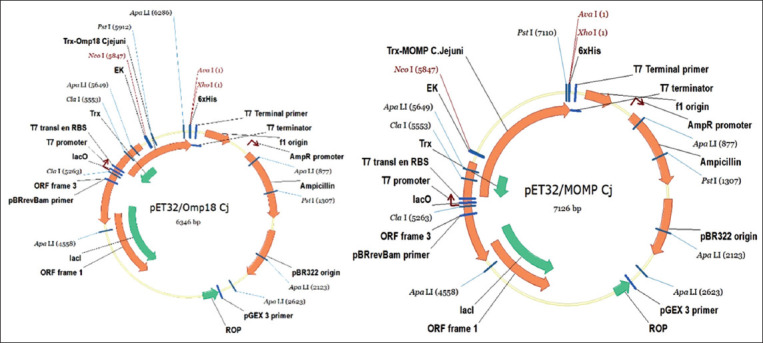
Schemes of genetic constructs carrying the genes for the major outer membrane protein and Omp18 proteins of *Campylobacter jejuni*.

The codon-optimized genes for the MOMP and Omp18 proteins were inserted into the pET32 vector. The pET32 vector was used because of its ease of expressing heterologous genes by inserting them in the sequence with the gene coding for thioredoxin protein, thereby increasing the solubility of the target protein. The expression of rOmp18 and rMOMP proteins in the genetic construct based on the pET28 vector was not observed. We, therefore, used the expression cassette of the plasmid vector pET32, which includes nucleic acid sequences coding for thioredoxin protein, His-Tag, S-Tag, thrombin, and enterokinase. His-and S-tags are generally utilized for protein isolation and purification, and the gene for 6His-Tag protein was used in this study for the expression and purification of MOMP and Omp18. The total molecular weights of the rOmp18 and rMOMP proteins are 36 kDa (Tag – 0.9 kDa, thioredoxin – 14, Omp18 – 18 kDa, thrombin – 4 kDa) and 64 kDa (Tag – 0.9 kDa, thioredoxin – 14 kDa, MOMP – 45 kDa, thrombin – 4 kDa), respectively (Figure-1).

*Escherichia coli* BL21(DE3) cells transformed with expression plasmids pET32/Omp18 and pET32/MOMP were cultured in LB medium with the appropriate antibiotic supplemented with IPTG at a concentration of 0.2 mM. At all-time points of material sampling, an increase in the gene expression of the Omp18 and MOMP proteins was observed. For the optimization of expression and the purification of the rMOMP and rOmp18 proteins, the transformed bacterial strains were cultured at various inductor concentrations (0.1, 0.2, 0.5, and 1 mM) and incubation temperatures of 25°C and 37°C. The expression of the recombinant proteins was evaluated by their relative content in the insoluble fraction (Table-1).

**Table-1:** Relative amounts of recombinant proteins (%) in insoluble fractions of lysates of producer strains under various conditions of expression induction.

Recombinant proteins	T°	IPTG concentrations			

		0.1 mM (%)	0.2 mM (%)	0.5 mM (%)	1 mM (%)
pET32/Omp18	25°C	20	10	10	5
	37°C	80	80	50	30
pET32/MOMP	25°C	20	10	10	5
	37°C	50	50	30	10

IPTG=isopropyl-β-D-1-galactopyranoside, MOMP=Major outer membrane protein

As the expression analysis showed, for all producer strains, the most optimal conditions for the expression of recombinant proteins are observed at 0.2 mM IPTG and 37°C. These conditions were therefore selected for further experiments.

**Figure-2:**
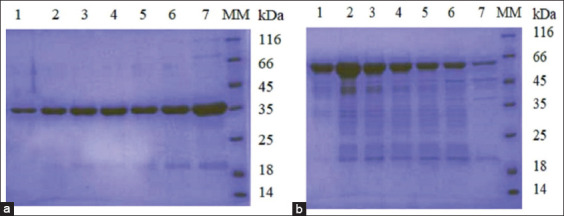
Sodium dodecyl sulfate-polyacrylamide gel electrophoresis of purified fractions of rOmp18 (a) and recombinant major outer membrane protein (b). Lane 1–7: purified recombinant protein fractions; MM: Molecular marker.

Following the standardized experimental procedure, purified preparations of antigens were obtained. The rOmp18 and rMOMP proteins were eluted using a buffer containing 200 mM imidazole (Figure-2).

### Liquid Chromatography with tandem mass spectrometry and western blot analysis of recombinant proteins

**Figure-3:**
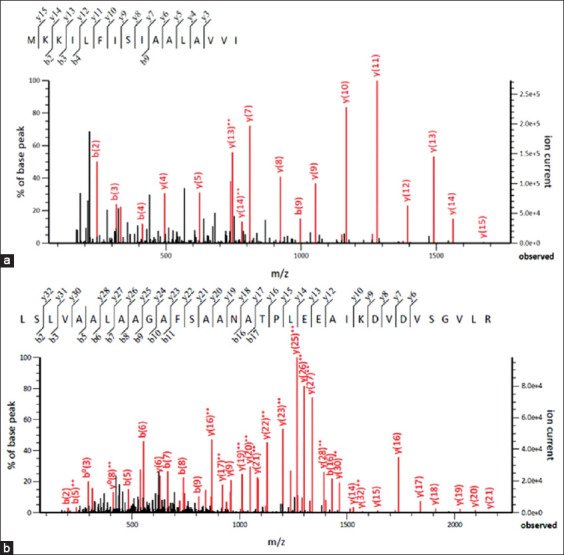
MS/MS spectra of trypsin-fragmented peptides of rOmp18 (a) and recombinant major outer membrane protein (b) antigens.

The recombinant proteins were analyzed by LC/MC-MS-spectrometry to determine their amino acid sequence. Peptides with the amino acid sequences MKKILFISIAALAVVI and LSLVAALAAGAFSAA NATPLEEAIKDVDVSGVLR were obtained. A search for the sequence of the obtained peptides in the SwissProt database matched the corresponding amino acid sequences to rOmp18 and rMOMP proteins of *C. jejuni* with a score of 18117 and 1610, respectively (Figure-3).

**Figure-4:**
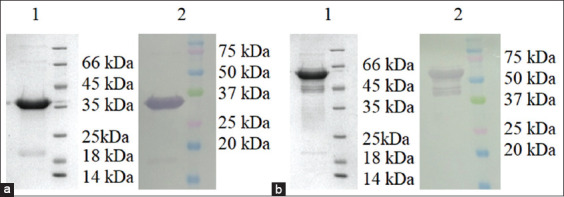
Sodium dodecyl sulfate-polyacrylamide gel electrophoresis (SDS-PAGE) and western blot of rOmp18 (a) and recombinant major outer membrane protein (b) with *Campylobacter jejuni* positive serum. Lane 1: SDS-PAGE; Lane 2: western blot.

Due to the use of high urea concentration during recombinant protein purification, there is a need for proper protein refolding. Refolding was carried out using a gradual decrease in the urea concentration and a parallel increase in imidazole concentration. To determine the quality of the refolding of recombinant proteins and the preservation of antigenic epitopes of the protein, western blotting was performed using control-positive sera. The analysis showed that the control-positive sera reacted specifically in western blotting, showing protein bands at 36 and 64 kDa, characteristic of rOmp18 and rMOMP, respectively (Figure-4).

### Enzyme-linked immunosorbent assay analysis of bovine serum pool

**Figure-5:**
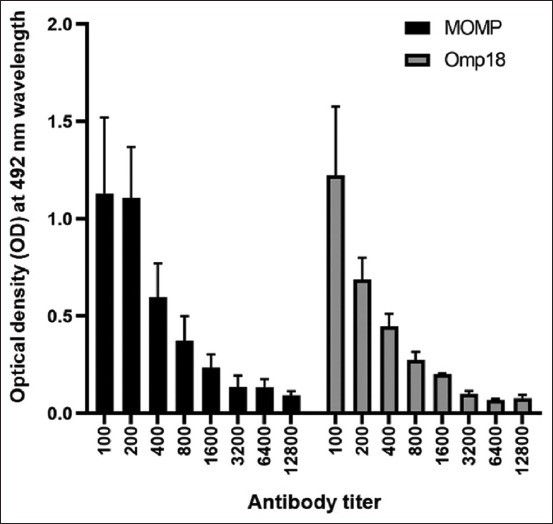
Titration histogram of six different dilutions of sera samples in enzyme-linked immunosorbent assay with recombinant major outer membrane protein and rOmp18 proteins. Error bars represent standard deviation.

The resulting rMOMP and rOmp18 proteins were used to study 94 sera samples from cows from farms where *C. jejuni* was isolated. The antibody titer was determined using ELISA in six randomly obtained serum samples in dilutions of 1:100–1:12800. As a negative control, bovine serum samples from campylobacteriosis-free farms were used. Enzyme-linked immunosorbent assay results showed the presence of antibodies against rOmp18 and rMOMP antigens in the serum, even at a dilution of 1:12800 (Figure-5).

**Figure-6:**
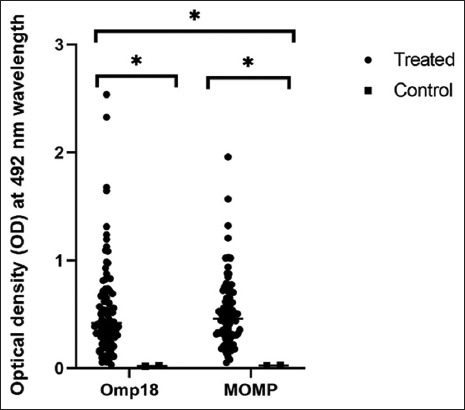
Optical density of the enzyme-linked immunosorbent assay reaction of recombinant major outer membrane protein and rOmp18 with bovine serum samples. Each symbol represents a separate serum sample. Statistical analysis was performed on the dataset using Student’s t-test: *p ≤ 0.05.

Analysis of the optical density of the reaction liquid of 94 sera at a dilution of 1:100 also showed the presence of significantly higher amounts of antibodies to rOmp18 and rMOMP antigens, and in some serum samples, a rather high concentration of antibodies was observed (Figure-6).

## Discussion

The objectives of this study were to obtain rOmp18 and rMOMP antigens and to evaluate their diagnostic properties. The first step was the design and production of genetic constructs coding for the genes of two diagnostic antigens, rOmp18 and Rmomp, from *C. jejuni*. The *Omp18* and *MOMP* gene sequences of *C. jejuni* were synthesized *de novo* with codons optimized for expression in *E. coli*. Finally, recombinant Omp18 and MOMP proteins with molecular weights of 36 and 64 kDa, respectively, were obtained and confirmed using specific antibodies.

*Campylobacter jejuni* is the main cause of gastrointestinal disorders of bacterial origin and is responsible for triggering many other diseases, such as Guillain-Barré syndrome, reactive arthritis, and other malignancies [18, 19]. However, the frequency of overlap of other infections with these diseases varies depending on the geographical location and the lack of standardized specific tests for *C. jejuni* further complicates diagnosis. The sensitivity and specificity of various tests can be disputed since antigens traditionally used in diagnostics are not always effective [13].

For efficient expression of recombinant proteins in *E. coli* BL21, the synthesized genes were cloned into the pET32 plasmid. The expression of the pET32/Omp18 and pET32/MOMP plasmids in the BL21 strain was high. The reading frame of the presented expression vectors includes, together with the thioredoxin gene, the recombinant protein gene and a hexahistidine tag, which allows for efficient purification of recombinant proteins using a HisTrap chromatographic column.

The linear decrease in the urea concentration of the HisTrap column during the purification of recombinant proteins allowed for the appropriate refolding of the desired proteins after their denaturation. The activity of the refolded protein was confirmed by western blotting and ELISA using positive-control sera. rOmp18 and rMOMP antigens were also found to be effective in the serological diagnosis of 94 blood serum samples obtained from infected cattle.

A similar approach was reported previously for the cloning of 14 *C. jejuni* genes into pET30, pET32a, and pGEX-4T-1 expression vectors for expression in *E. coli* BL21. Protein purification was performed using histidine (His) and glutathione S-transferase tags. Evaluation of the antigenicity of the proteins by western blotting and ELISA with rabbit immune sera showed the potential utility of the antigens in the serological diagnosis of *C. jejuni* [20]. Among the proteins listed above, Omp18 was also studied, which differs from our Omp18 protein in two amino acids at positions 7 (Thr → Ile) and 78 (Thr → Ala).

Burnens *et al*. [4] cloned and expressed, in *E. coli*, the gene encoding the immunogenic Omp18 protein from the reference *C. jejuni* strain (ATCC 29428). The analysis of the recombinant protein in various immunological reactions showed that the rOmp18 protein was an effective tool for the serological diagnosis of campylobacteriosis. Using specific mAbs, the authors determined the localization of the Omp18 antigen on the outer membrane of the pathogenic *C. jejuni*. These results confirm the efficacy of rOmp18 and specific mAbs in pathogen identification in food products obtained from animal sources. In contrast to the described work, we used the pET32 vector, resulting in a protein molecular weight of 36 kDa due to the insertion of thioredoxin, a His-tag, and a thrombin. The presence of thioredoxin increases the solubility of the recombinant protein.

Ferrara *et al*. [21] successfully performed recombinant expression, induction, and purification of the recombinant *C. jejuni* 85H MOMP protein in an *E. coli* expression system. The structure and function of the recombinant MOMP protein expressed in *E. coli* did not differ from the native MOMP obtained from *C. jejuni*. Interestingly, the 18-strand β-chain MOMP of the *C. jejuni* antigen showed the same conformational structure as OmpF and OmpC, which are 16-strand β-chains.

In western blot and ELISA, rOmp18 and rMOMP antigens reacted specifically with positive-control antibodies. When examining a pool of sera from infected cattle, analysis by ELISA using the recombinant proteins showed high sensitivity and 90% specificity.

It should be noted that the results obtained in our can only be used for serological diagnosis of animal campylobacteriosis. In the future, mAbs of these antigens need to be obtained to identify pathogens present in food products and pathological samples.

## Conclusion

At present, recombinant antigens are widely used in developing diagnostic test systems for public health and veterinary medicine. While the expression of recombinant antigens of infectious agents, in *E. coli*, has been performed intensively, purification of recombinant antigens expressed in *E. coli* presents some difficulties due to aberrant protein folding. For the formation of appropriate conformational structures of recombinant proteins, the most effective method is the linear decrease of the urea gradient on HisTrap columns. This method made it possible to correctly generate recombinant *C. jejuni* Omp18 and MOMP antigens, which specifically reacted with positive-control sera. This study demonstrated the suitability of *C. jejuni* rOmp18 and rMOMP antigens in the serological diagnosis of campylobacteriosis.

## Authors’ Contributions

SB and KT: Design and management of the study. KT and AS: Materials and methods preparation. KT, AS: Generation of genetic constructs, expression, and purification of recombinant proteins. AB and AZ: Bovine sera sampling and ELISA analysis. SB: Data analysis. SB and KT: Wrote and edited the manuscript. All authors have read and approved the final manuscript.
